# Photocatalytic Micro/Nanomotors Functioning in the Near‐Infrared Window for Biomedical Applications

**DOI:** 10.1002/advs.202522483

**Published:** 2026-02-11

**Authors:** Yufen Chen, João Marcos Gonçalves, Katherine Villa

**Affiliations:** ^1^ Institute of Chemical Research of Catalonia (ICIQ) The Barcelona Institute of Science and Technology (BIST) Tarragona Spain; ^2^ Institució Catalana de Recerca i Estudis Avançats (ICREA) Barcelona Spain

**Keywords:** bio‐application, defect engineering, heterojunctions, NIR light, photocatalytic micro/nanomotors, upconversion nanoparticles

## Abstract

Near‐infrared (NIR) light‐driven photocatalytic micro‐ and nanomotors are emerging as a new class of self‐propelled micro/nanodevices for minimally invasive biomedical applications. By operating within the biological transparency window, they enable autonomous motion and light‐induced redox reactions under biocompatible illumination conditions. Yet, the low photon energy of NIR light imposes fundamental constraints on photocatalytic efficiency and propulsion, requiring innovative materials design. This review systematically discusses recent progress in materials strategies for achieving NIR responsiveness, including heterostructure formation, upconversion coupling, defect modulation, and photosensitization via dyes or plasmonic nanostructures. The relationships between material composition, optical absorption, charge separation, and motion behavior are analyzed, with emphasis on photocatalytic propulsion. Particular attention is given to their potential application in photodynamic therapy, neural stimulation, and redox‐based treatments, while discussing remaining challenges related to fuel‐free propulsion, ionic tolerance, and immune system evasion. Finally, key design principles and future research directions are outlined, positioning NIR‐responsive photocatalytic micro/nanomotors as a versatile platform for minimally invasive therapeutic treatments and remote‐controlled catalysis.

## Graphical abstract

This review focuses on near‐infrared responsive photocatalytic micro/nanomotors based on inorganic materials, with particular emphasis on minimally invasive biomedical applications. It discusses material strategies for achieving near‐infrared responsiveness, including heterostructure construction, upconversion nanoparticle integration, defect engineering, and photosensitization techniques. The review further highlights emerging therapeutic applications, key challenges and opportunities, and future design principles.

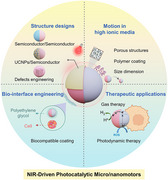

## 1 Introduction

Light‐driven technologies have significantly advanced the field of biomedicine, enabling high‐resolution imaging and therapeutic strategies with exceptional spatiotemporal precision [1, 2, 3]. Recent developments suggest that integrating photoactive materials with autonomous, self‐propelled micro/nanoscale devices (namely micro/nanomotors), holds great promise for enhancing targeted drug delivery, reducing invasiveness, and improving the overall efficiency of therapeutic treatments [4, 5, 6]. Notably, light‐driven photocatalytic micro/nanomotors capable of converting light energy into mechanical motion have opened new avenues not only in biomedicine but also in environmental remediation and energy conversion [6, 7, 8]. While most studied photocatalytic micromotors rely on UV or visible light absorption, extending their photoactivation into the NIR window (700–1700 nm) unlocks major opportunities for biomedical use [9, 10, 11].

NIR photons offer deep tissue penetration with minimal photodamage, enabling remote actuation in physiological environments. Unlike NIR‐driven thermophoretic micro/nanomotors, which require high light intensities to induce thermal gradients [12, 13, 14, 15, 16, 17], NIR‐driven photocatalytic micro/nanomotors rely on localized surface redox reactions to generate photochemical gradients, leading to their autonomous motion. This redox photoactivation mechanism enables propulsion under low, biologically compatible light intensities and in the presence of naturally available chemical species. Moreover, the unique ability of photocatalytic micro/nanomotors to catalyze in situ chemical reactions that generate reactive oxygen species (ROS) and hydrogen makes them especially attractive for redox‐based therapeutic approaches [18, 19]. However, the lower photon energy of NIR light also limits photocatalytic reactivity of photo‐responsive materials, posing fundamental challenges to achieve effective motion. Recent advances in semiconductor engineering adopt strategies such as bandgap tailoring, heterojunction structure fabrication, and surface modification to enhance NIR absorption capabilities while maintaining robust redox performance and photostability. Therefore, understanding how these material parameters govern propulsion remains central to the development of high‐performance NIR‐driven photocatalytic micro/nanomotors.

Unlike previous reviews that broadly survey various types of photoactive micro/nanomotors, including photothermal‐driven, visible light‐response, or optoelectronic tweezers [7, 20, 21, 22], this work uniquely concentrates on NIR‐responsive photocatalytic micro/nanomotors based on inorganic materials. In addition, it clearly differentiates photocatalytic propulsion from photothermal effects in previously reported NIR light‐driven systems, contributing to a better understanding of mechanistic aspects and associated challenges. By addressing key bottlenecks in photocatalytic micro/nanomotors, including limited propulsion efficiency of UV/visible‐light‐driven systems for bioapplications, challenges in achieving efficient and stable NIR‐driven designs, and poor performance in physiological environments, this review fills a critical gap in mechanistic analyses of NIR‐driven photocatalytic micro/nanomotors and provides fundamental insights to guide future research. Specifially, it examines the fundamental principles governing NIR light–matter interactions and the essential materials design strategies that enable photocatalytic propulsion, such as heterojunction formation, bandgap engineering, integration of upconversion nanoparticles (UCNPs), and decoration with photosensitizers (PSs). Furthermore, this review also discusses emerging biomedical applications that exploit NIR transparency and photocatalytic redox activity, offering new opportunities for advanced treatments, such as neural modulation and photoredox cancer therapy. Finally, we examine the challenges associated with operating in biological environments and outline future research directions toward clinically relevant, light‐programmed photocatalytic micro/nanodevices.

## 2 Unique Properties of NIR Light Irradiation

Light plays a fundamental role in numerous natural processes, from driving photosynthesis in plants to regulating biological rhythms in living organisms [23, 24]. Fundamentally, these processes arise from light‐matter interactions, dictated by the energy of individual photons. According to Equation 1, the photon energy is directly proportional to its frequency and inversely proportional to its wavelength:

(1) $$E=\frac{hc}{\lambda }$$

where *E* is the photon energy, *h* is Planck's constant, *c* is the speed of light, and λ is the wavelength of the light radiation.

The absorption wavelength threshold (*λ_g_*) of a semiconductor is intrinsically linked to its bandgap energy (*E_g_*), making this relationship a key design parameter for light‐driven photocatalytic micro/nanomotors. Specifically, shorter wavelengths correspond to higher‐energy photons that can excite wide bandgap semiconductors, whereas narrow bandgap materials respond to longer wavelengths, such as NIR light. Thus, bandgap tailoring is essential for extending photoactivation into the desired spectral region.

**FIGURE 1:**
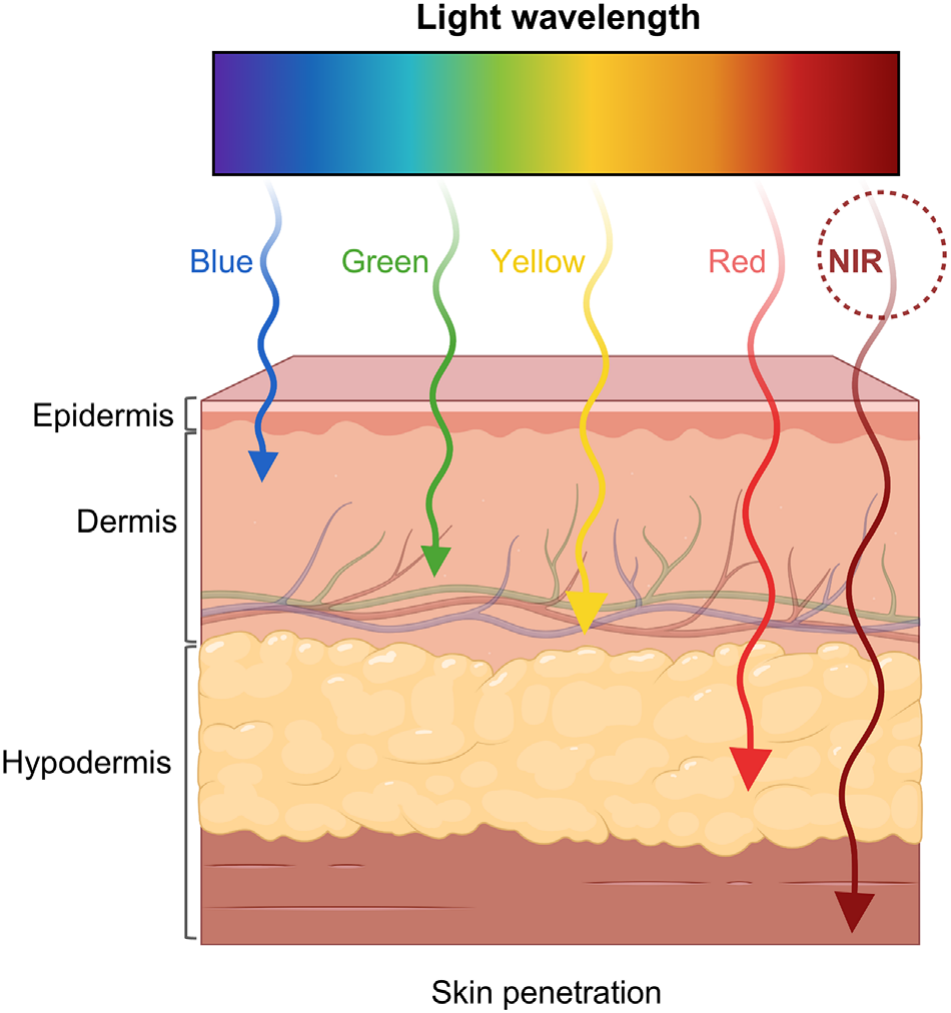
Penetration of light at different wavelengths through biological tissue, showing minimal absorption and scattering within the NIR transparency window.

Beyond the intrinsic semiconductor properties, the optical characteristics of light, including penetration depth, energy input, abundance, and biocompatibility, also influence photocatalytic performance and determine suitability for different applications. Figure 1 illustrates the penetration of light of various wavelengths through biological tissue. For instance, UV light possesses high photon energy but represents only ca. 4% of the solar spectrum and induces cytotoxic effects on skin and other tissues, greatly limiting its biomedical applicability [25]. Visible light, while more abundant, suffers from limited tissue penetration, restricting its use to superficial skin therapies. In particular, blue light, with relatively high photon energy, has demonstrated efficacy in dermatology and antimicrobial treatments [26, 27], but remains ineffective for deeper penetration regions.

In contrast, NIR light can safely reach subdermal regions with minimal absorption by biological tissues and substrates [28], delivering light doses with precision and uniformity [29, 30]. In general, NIR light can be divided into NIR‐I and NIR‐II based on their respective wavelength range. The region spanning 750–950 nm constitutes the first NIR window (NIR‐I), a spectral range in which biological tissues exhibit relatively high transparency. Light in the second NIR window (NIR‐II, 1000–1700 nm) enables deeper tissue penetration and allows for a higher maximum permissible exposure (MPE), thereby improving phototherapeutic performance [31, 32]. This deeper penetration is particularly advantageous for photodynamic therapy (PDT), where the effective light dose depends on how far the irradiation can propagate within tissue. Penetration depth increases steadily across the NIR spectrum, reaching approximately 1.5, 2.0, 2.3, and 2.5 mm at 600, 700, 800, and 900 nm, respectively [33]. It has been reported that high doses of NIR exposure (above 4000 J/cm^2^) are necessary to induce photodamage in bacteria and fungi [34]. A recent work using a three‐dimensional (3D) skin model further demonstrated wavelength‐dependent variations in cellular viability. To achieve comparable levels of photodamage, UV light required only 30 J/cm^2^, blue light demanded 60 J/cm^2^, whilst NIR necessitated 540 J/cm^2^ [35]. This clearly demonstrate the distinct advantage of NIR compared to other wavelength ranges. This combination of deep penetration, low phototoxicity, and spectral compatibility with emerging nanomaterials defines NIR light as an optimal driver for photocatalytic micro/nanomotors.

## 3 Design Principles for NIR‐Responsive Photocatalytic Micro/Nanomotors

**FIGURE 2:**
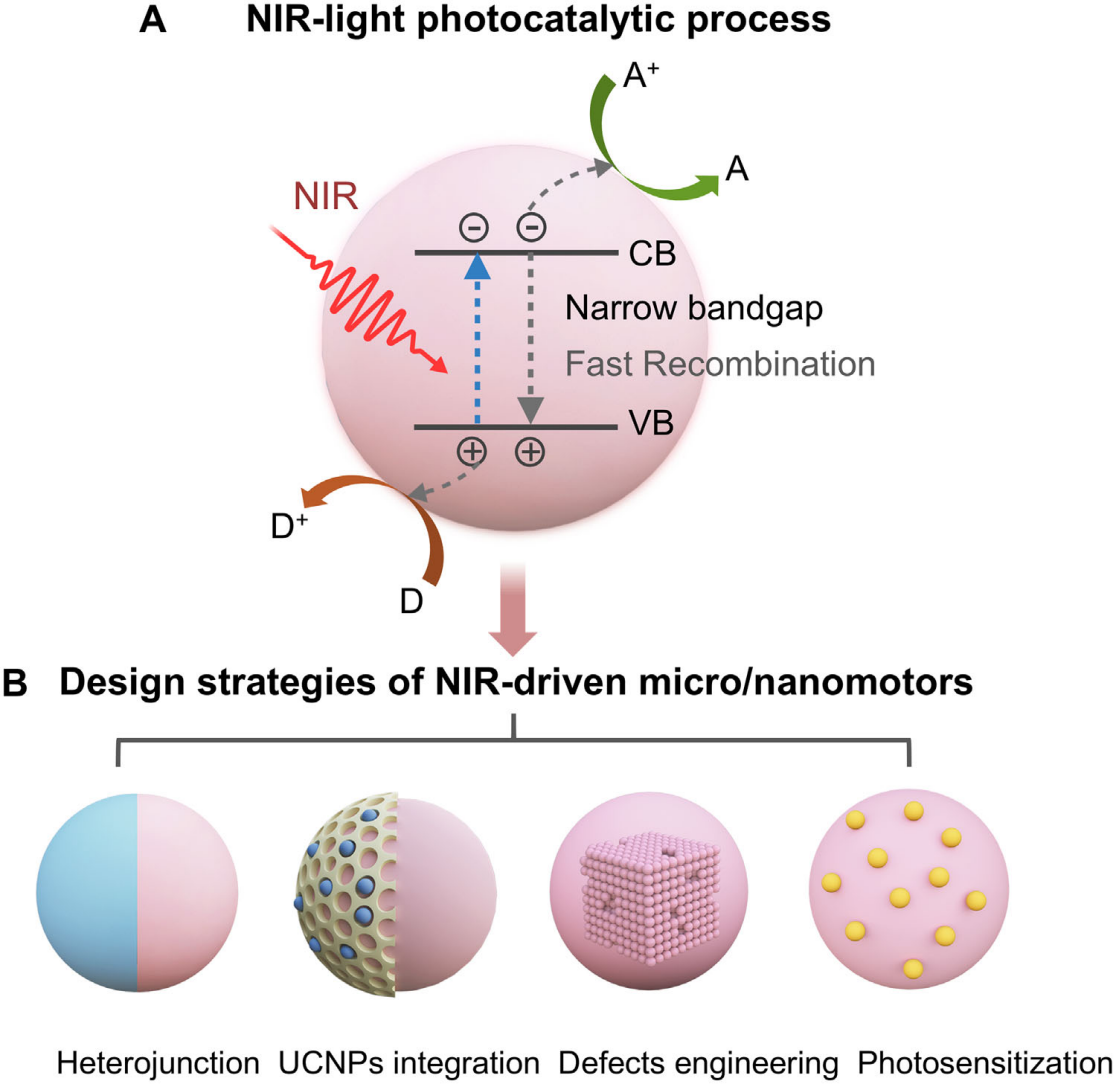
Key principles and material strategies for NIR‐driven photocatalytic micro/nanomotors discussed in this review. (A) Schematic illustration of the photocatalytic mechanism. (B) Corresponding design strategies that enable efficient light‐to‐motion conversion. UCNPs: upconversion nanoparticles.

The activation mechanism of NIR light‐driven photocatalytic micro/nanomotors is fundamentally analogous to that of UV‐ and visible‐light‐responsive systems, relying on asymmetrical structures to achieve autonomous motion [36]. Upon NIR illumination, photons excite the semiconductor, generating electron–hole pairs that subsequently undergo separation, migration, and/or recombination processes (Figure 2A). Such photogenerated charge carriers drive localized surface redox reactions, producing asymmetric chemical gradients across the micro/nanomotor surface. The resulting imbalance in reaction products gives rise to a net photocatalytic propulsion force. Since the general fabrication strategies and detailed propulsion mechanisms of light‐driven micro/nanomotors have been thoroughly reviewed elsewhere [12, 36, 37, 38], the present review focuses on design principles, material integration, and surface engineering strategies that enable efficient NIR‐responsive photocatalytic propulsion, with an emphasis on biomedical applicability.

Achieving efficient motion under NIR illumination requires photocatalytic materials that combine the following key characteristics: i) strong NIR light absorption, typically achieved through narrow bandgaps or auxiliary absorbers; ii) efficient charge separation and rapid migration of photogenerated carriers to minimize recombination losses; iii) appropriately aligned conduction and valence band (CB and VB, respectively) edges that provide sufficient driving force for the targeted redox reactions; and iv) a high surface density of catalytically active sites to accelerate interfacial reactions and improve overall conversion efficiency [39].

Narrow‐bandgap semiconductors, typically in the range of 1.3–2.2 eV, satisfy the first requirement, as they can absorb photons within the NIR region. Representative examples include Ag_2_O (1.3 eV) [40], MoS_2_ (1.2 eV) [41], Ag_2_Te (0.67) [42], WS_2_ (1.4 eV) [43], CdSe (1.70 eV) [44], BiOI (1.7–1.9 eV) [45], black phosphorus (BP, 0.3–2.1 eV), among others [46]. However, the use of narrow‐bandgap materials often entails rapid charge recombination, which drastically lowers photocatalytic efficiency. To mitigate this drawback, coupling these materials with wide‐bandgap semiconductors is a promising strategy to facilitate the interfacial charge separation efficiency, thereby enabling more effective chemical conversion and light‐driven propulsion. However, the integration of NIR‐responsive materials into micro/nanomotor architectures remains in its infancy, calling for rational design approaches that unify multiple functional capabilities.

In this section, we discuss current synthetic strategies that address narrow‐bandgap limitations, including the construction of semiconductor heterojunctions and hybrid architectures incorporating UCNPs to broaden light absorption, as well as complementary approaches such as defect engineering and photosensitization using molecular dyes or plasmonic nanostructures (Figure 2B). These strategies outline a roadmap toward the realization of highly efficient photocatalytic micro/nanomotors, capable of NIR‐driven propulsion without the need for thermoresponsive components. Representative examples of NIR‐photocatalytic micro/nanomotors reported to date are summarized in Table 1.

**TABLE 1:** Reported NIR‐photocatalytic micro/nanomotors. The abbreviations used in this table (from top to bottom): GO: Graphene oxide, PDA: Polydopamine, TPM: 3‐(Trimethoxysilyl)propyl methacrylate, OSC: Organic semiconductor solar cell, BQ: 1,4‐benzoquinone, H_2_Q: Hydroquinone.

Micromotors	Size	Wavelength	NIR light intensity	Fuel (concentration)	Motion velocity	Propulsive force	References
WS_2_/Pt/Ni	Length: 11.4 ± 1.1 µm	800 nm	N/A	H_2_O_2_ (0.5%)	94 µm/s	Bubble propulsion	[47]
Cu_2_O@GO	Diameter: 1.5–2 µm	808 nm	3.2 W/cm^2^	Glucose, leucine, and urea (50 mM of each fuel)	11.39, 6.16, 5.51 µm/s, respectively	Synergistic effects of photothermal effects and photocatalysis	[48]
Cu_2_O@PbS	Diameter: 1.3 µm	808 nm	2 W/cm^2^	Malic acid (0.125 mM)	11.86 µm/s	Concentration gradient of photocatalytic products	[49]
Au‐Ni‐PDA‐CuS	Length: 11.46 µm	808 nm	1.8 W/cm^2^	H_2_O	2.06 µm/s	Photocatalytic reaction	[50]
TPM/Ni/OSC/Au	Microsphere, diameter: 2 µm	808 nm	70 mW/cm^2^	BQ/H_2_Q (N/A)	14.3 µm/s	Photoelectrochemical reaction‐induced self‐diffusiophoresis	[51]

### 3.1 Current Strategies

#### 3.1.1 Heterostructured Micro/Nanomotors

Heterostructures, formed at the interface between two or more materials with complementary redox properties, have emerged as a highly effective strategy for enabling the motion of photocatalytic micro/nanomotors under NIR light irradiation. These systems typically involve semiconductor‐metal, and semiconductor‐semiconductor junctions. By engineering appropriate band alignments, the heterostructures can significantly improve the photostability and overall photocatalytic activity [52, 53]. Such enhanced performance is critical for the sustained operation of micro/nanomotors based on narrow bandgap semiconductors, which are inherently prone to photocorrosion under prolonged light irradiation [54]. Moreover, the formation of heterojunctions allows for the precise tuning of redox potentials through CB and VB alignment, enabling the rational design of micro/nanomotors tailored to specific applications. Up to date, only Cu_2_O has been successfully used in the development of NIR‐driven photocatalytic micromotors for different biomedical applications [55]. In contrast, other promising narrow‐bandgap materials, such as MoS_2_ [56], BiOI [57], CdS and CdSe have so far been investigated exclusively in visible light‐responsive micro/nanomotors [58, 59].

This gap evidences a significant opportunity for further research, as many suitable photocatalytic materials remain unexplored for the development of more efficient NIR‐driven micro/nanomotors. However, expanding the material selection must be balanced with careful consideration of biocompatibility, particularly for in vivo applications. For example, CdS‐based systems can release cadmium ions into the cellular environment, which induces cytotoxic effects. Although generally undesirable, this toxicity has been intentionally exploited in cancer therapy and antimicrobial applications [60]. In this context, plant extract‐mediated CdS quantum dots have demonstrated excellent biocompatibility, suggesting their potential for the fabrication of safer photocatalytic micro/nanomotors [61].

##### 3.1.1.1 Semiconductor‐Metal

Prior to the development of NIR‐driven photocatalytic micro/nanomotors, early studies have demonstrated the effectiveness of semiconductor–metal heterojunctions in achieving light‐driven propulsion under red light (590–650 nm) irradiation. Owing to its deeper tissue penetration and reduced phototoxicity compared to UV light, red light‐driven micro/nanomotors are particularly suitable for skin‐related treatments, such as PDT and wound healing [62, 63]. A representative example is the Janus black titania (Au/TiO_2_) micromotor, which incorporates boron‐doped TiO_2_ and a gold half‐coating. The movement of as‐prepared Au/TiO_2_ micromotors under red light was attributed to their relatively narrow bandgap of 1.75 eV [64]. However, its propulsion still required the presence of 3 wt.% H_2_O_2_, limiting its practicality for therapeutic applications. When the concentration of H_2_O_2_ exceeds the level that an organism's antioxidant system can effectively dispose (ca. 50–100 µM), it will damage DNA, lipids and proteins [65, 66]. To extend this concept into the NIR region, a WS_2_/Ni/Pt tubular micromotor was developed, incorporating a semiconductor–metal heterojunction responsive to NIR irradiation [47]. Nevertheless, its motion mechanism still relied on Pt‐catalyzed chemical fuel decomposition, a dependence that continues to hinder progress toward truly fuel‐free operation required for bio‐applications.

##### 3.1.1.2 Inorganic Semiconductor‐Inorganic Semiconductor

**FIGURE 3:**
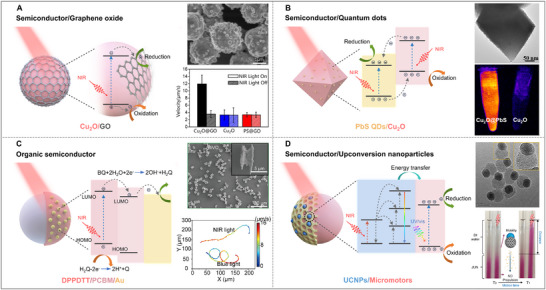
Schematic illustration of the representative material architectures and the corresponding reported examples enabling NIR‐responsive photocatalytic micromotors. (A) Micromotor based on a semiconductor/photothermal material configuration: Cu_2_O/GO. Adapted with permission.[48] Copyright 2021, Elsevier. (B) Micromotor based on a semiconductor/quantum dots (QDs) heterostructure: Cu_2_O@PbS QDs. Adapted with permission.[49] Copyright 2022, American Chemical Society. (C) Micromotor based on the combination of organic semiconductors: BiVO_4_/PCBM/DPPDTT. Adapted with permission.[67] Copyright 2026, John Wiley and Sons. (D) Micromotor based on a semiconductor/upconversion nanoparticles (UCNPs) design: mSiO_2_/UCNP/RB. Adapted with permission.[68] Copyright 2024, John Wiley and Sons. BQ: 1,4‐benzoquinone, H_2_Q: hydroquinone, DPPDTT: diketopyrrolopyrrole, PCBM: [6,6]‐Phenyl C_61_ butyric acid methyl ester.

One of the earliest examples of purely photocatalytic micromotors operating under NIR light involved heterostructured silicon nanomotors (n^+^‐Si/p‐Si), demonstrating self‐propulsion when irradiated with 808 nm light [69]. In addition, Cu_2_O, a p‐type semiconductor with a slightly larger bandgap of 2.1 eV, has become a benchmark material for designing NIR‐responsive micro/nanomotors due to its efficient NIR light‐harvesting properties and relatively simpler synthesis. Cu_2_O is not only non‐toxic but also theoretically capable of performing water splitting, based on the favorable positions of its CB and VB. Cu_2_O exhibits limited photostability and often undergoes oxidation to CuO or reduction to metallic Cu during photoactivation; however its light‐induced oxidation to CuO can be strategically utilized as a source of Cu^2+^ ions for effective anticancer and antifungal applications [70, 71, 72]. Nevertheless, issues such as uncontrolled phase transformation and particle agglomeration still remain major obstacles to its reliable bioactive use. Therefore, incorporating Cu_2_O into heterostructures is essential to enhance its stability and preserve its photocatalytic activity. A major advancement in the field came in 2021 with the development of Cu_2_O micromotors coated with graphene oxide (GO), which self‐propelled under NIR light irradiation using biocompatible fuels. The photocatalytic mechanism under NIR light is shown in Figure 3A [48]. Upon NIR photoactivation, electrons in the CB of Cu_2_O transferred to GO and further engaged in the subsequent reduction reactions, while the holes remained in Cu_2_O for the oxidation reactions. Such strategy largely improved the electron‐hole separation and prevented photocorrosion. Consequently, the resulting micromotors were able to self‐propel in glucose, leucine, and urea solutions. The propulsion force was attributed to a synergistic effect of photocatalytic activity of Cu_2_O and photothermal properties of GO. On the one hand, the high temperature induced by GO accelerated the photocatalytic reaction; on the other hand, it enhanced product diffusion velocities and fluid transport around the motor, thereby generating stronger propulsive force. Furthermore, this work also demonstrated that propulsion originated primarily from Cu_2_O rather than GO, as evidenced by the performance of GO modified polystyrene microspheres tested under the same experimental conditions. The polystyrene@GO particles displayed the same speed as pristine Cu_2_O micromotors, suggesting that the importance of Cu_2_O as electron donor in this system (Figure 3A). Interestingly, when GO was replaced with PbS quantum dots (QDs, Figure 3B), the Cu_2_O@PbS micromotors not only showed propulsion under 808 nm NIR light but also exhibited intrinsic fluorescence at 1100 nm (NIR‐II), making them valuable for real‐time tracking and imaging [49]. Besides, recent reports have also described that CuS‐based tubular micromotors, Au‐Ni‐polydopamine (PDA)‐CuS, displayed propulsion under 808 laser irradiation [50]. However, since PDA was also integrated into this system, potentially introducing photothermal effects, it is challenging to isolate the contribution of photocatalytic propulsion from the observed motion. Therefore, when integrating photothermal materials into micro/nanomotor designs, appropriate control experiments are essential to disentangle the respective contributions of photocatalytic and photothermal effects.

##### 3.1.1.3 Inorganic Semiconductor‐Organic Semiconductor

Organic semiconductors render a promising alternative to traditional inorganic photocatalysts in heterojunction configurations, providing unique advantages, including ease of fabrication, improved batch‐to‐batch consistency, and broad spectral responsiveness, with efficient performance under both visible and NIR light [73, 74]. When combined with inorganic semiconductors, the resulting hybrid systems can enhance light absorption, charge separation, and overall photocatalytic efficiency.

A recent study investigated the design of NIR‐driven micromotors using materials commonly found in photovoltaic cells. Specifically, a heterojunction mixture of [6,6]‐Phenyl C_61_ butyric acid methyl ester (PCBM) and diketopyrrolopyrrole (DPPDTT) was employed, where PCBM acted as the electron acceptor and DPPDTT as the electron donor, leading to enhanced charge separation and increased photocatalytic efficiency, as shown in Figure 3C [51]. These organic materials were simply deposited by spin‐coating onto micromotors with various geometries, such as zero‐dimensional (0D), one‐dimensional (1D), two‐dimensional (2D), and 3D, demonstrating the versatility of this approach for fabricating micromotors across different morphologies. Subsequently, butterfly‐shaped BiVO_4_ micromotors coated with PCBM and DPPDTT were fabricated through the same protocol, achieving dual activation under blue and 808 nm NIR light within a single Janus architecture [67], as shown in Figure 3C. In addition to these examples, other classes of organic semiconductors, such as phthalocyanines (Pcs), also hold great promise for the development of NIR‐responsive systems. In an early study, Domen et al. demonstrated that the magnesium phthalocyanine (MgPc) loaded mesoporous g‐C_3_N_4_ exhibited NIR response at ca. 820 nm for H_2_ evolution [75]. This work opens up the possibility of combining Pcs with inorganic photocatalysts as a viable strategy for constructing NIR‐activated micro/nanomotors.

#### 3.1.2 Upconversion‐Integrated Micro/Nanomotors

Another effective strategy to extend photocatalytic propulsion into the NIR region involves integrating UCNPs with inorganic semiconductors (Figure 3D). UCNPs convert low‐energy NIR photons into higher‐energy visible or UV emission, which can subsequently drive photocatalytic reactions in the coupled semiconductor. To better understand how this combination enhances micro/nanomotor performance, we first outline the fundamental upconversion mechanisms, then discuss representative dopants and host materials, and finally examine their integration into micro/nanomotor systems along with emerging design opportunities.

**FIGURE 4:**
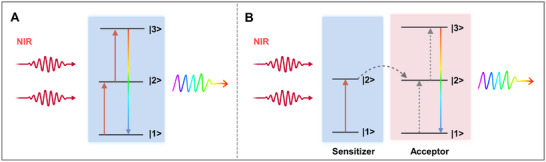
Most common mechanisms for upconversion process. (A) ESA, involving a single ion, and (B), ETU, involving two ions. ESA: excited‐state absorption, ETU: energy transfer upconversion.

Upconversion is a nonlinear optical phenomenon that enables anti‐Stokes emission, in which multiple low‐energy excitation photons (typically in the NIR) are absorbed sequentially and re‐emitted as a photon of higher energy (commonly in the visible range) [76, 77, 78]. In lanthanoid‐doped materials, upconversion emission typically occurs through excited‐state absorption (ESA) within a single dopant ion, or energy transfer upconversion (ETU) between a sensitizer and an activator (or acceptor) ion, as illustrated in Figure 4. In ESA, the dopant absorbs one excitation photon, populating its intermediate level |2>. If such dopant exhibits a sufficiently long lifetime, a second photon can be absorbed, leading to the population of the emitting level |3>, which prompts the radiative decay |3> → |1>, emitting a single high‐energy photon (Figure 4A). A similar process occurs with ETU: firstly, a sensitizer ion absorbs the incident photon and transfer the energy to a resonant excited state of a neighbouring acceptor populating the |2> state of the activator ion. As a result, two possibilities emerge: either the sensitizer can absorb a second photon and transfer the energy to the acceptor, or the latter absorb the second photon, both leading to the population of the |3> excited state and subsequent emission of higher energy photons (Figure 4B) [76, 77, 79]. It is worth noting that these are simplified mechanisms, and often non‐radiative transitions are also involved [79].

Among the various upconverting materials, those doped with trivalent lanthanoid ions (Ln^3^^+^) are the most widely used, due to their unique photophysical characteristics that make them particularly well‐suited for the energy conversion process. These features include: i) a rich energy level structure with excited states resonant with commercially available laser excitation wavelengths, and ii) long‐lived excited states, which allow for the accumulation of excitation energy from successive photon absorption events [76, 79, 80, 81]. Moreover, their low toxicity, high resistance to photobleaching, good biocompatibility and autofluorescence make UCNPs attractive light transducer for diagnosis and therapy in biomedicine [82, 83, 84]. The most common dopant pairs, Yb^3+^‐Er^3+^ and Yb^3+^‐Tm^3+^, generate green‐red and blue emission, respectively, while other lanthanoid ions or more complex core@shell@shell architectures enable further tuning of the emission across different wavelengths [85, 86, 87, 88, 89, 90, 91]. Efficient upconversion further depends on host lattices with low phonon energies, which suppress non‐radiative relaxation and enhance quantum yield. Some common hosts include fluorides [92, 93], molybdates [94], phosphates [95], and bismuth vanadate [96].

When integrated into micro/nanomotors, UCNPs commonly serve both functional and diagnostic roles, enabling simultaneous sensing and imaging. For instance, Yb^3+^‐Er^3+^ dopants have been used to impart green emission in BiVO_4_ micromotors [97], enable temperature sensing [98, 99], and sensitize organic molecules for therapeutic applications [100, 101]. In contrast, Yb^3+^‐Tm^3+^ dopants have been used as sensors for fuel‐decomposition products [102], and embedded into covalent organic frameworks (COFs) for optoacoustic in vivo imaging [103].

Although most reported systems exploit UCNPs for optical functionalities rather than propulsion, a few examples demonstrate direct upconversion‐driven motion. In one case, NaYF_4_:Yb^3+^,Er^3+^ conjugated with tetra(4‐carboxyphenyl)porphyrin iron(III) enabled H_2_O_2_ decomposition under 980 nm excitation, providing propulsion forces to millimetre‐sized motors [104]. More recently, Janus NaYF_4_:Yb^3+^,Tm^3+^‐mesoporous SiO_2_ micromotors loaded with a photosensitive Roussin's black salt produced nitric oxide upon salt photolysis via upconverted emission, resulting in self‐propulsion, as shown in Figure 3D [68]. These studies not only confirm the feasibility of UCNP‐based NIR actuation but also evidence the need for higher NIR‐to‐visible conversion efficiency and more effective integration within self‐propelled photocatalytic systems.

Some potential strategies include tuning dopant‐host combinations to match the UCNP emission wavelength with the semiconductor bandgap. For example, Yb^3+^‐Er^3+^‐doped UCNPs can sensitize visible‐light‐driven micromotors (e.g., BiVO_4_ and Cu_2_O), whereas Yb^3+^‐Tm^3+^‐doped UCNPs are better suited for UV‐responsive systems (e.g., TiO_2_ and ZnO). In addition, the development of UCNPs excitable at alternative NIR wavelengths (such as 808, 1064 or 1300 nm) could reduce water absorption and photothermal heating, enhancing their biocompatibility for in vivo use. Collectively, these advances will enable the creation of hybrid UCNP/photocatalytic micro/nanomotors that combine propulsion, sensing and therapeutic functions under biologically safe NIR illumination.

In summary, although all the strategies discussed above show strong potential for enabling NIR‐driven photocatalytic propulsion, a careful assessment of their respective advantages and limitations remains essential. For instance, inorganic semiconductor‐metal heterojunctions offer high propulsion efficiency and morphological homogeneity, but their low synthesis yield and reliance on toxic chemical fuels limit broader applicability. In contrast, heterojunctions composed of two inorganic semiconductors may provide better scalability, yet they introduce toxicity concerns associated with unstable materials, such as CdS. A complete overview of each discussed strategy is presented in Table 2.

**TABLE 2:** Key advantages and drawbacks of current strategies for improving photocatalytic self‐propulsion in NIR region.

Semiconductor‐based heterojunction	Advantages	Drawbacks
Inorganic‐Metal	High propulsion efficiency Uniform morphology	H_2_O_2_ as fuel Low material synthesis yield
Inorganic‐Inorganic	Biocompatible fuels Material versatility Scalability	Concerns about toxicity Low propulsion efficiency
Inorganic‐Organic	Material versatility High motion velocities Scalability	Relies on HQ/BQ Stability in physiological conditions Photobleaching
UCNPs‐Inorganic	Excitation wavelength versatility Resistant to photobleaching	Low quantum yields Relies on H_2_O_2_ or Roussin's black salt

### 3.2 Emerging Strategies for Enhanced NIR‐Light Responsiveness

#### 3.2.1 Defect Engineering

While narrowing the bandgap is a common strategy to enhance light absorption in the NIR region, it can also compromise the redox potentials required to drive the desired chemical reactions. Therefore, it is critical to maintain a balance between optimizing light harvesting and maintaining sufficient redox power [30]. One viable approach is introducing mid‐gap states into wide‐band semiconductors, an area where defect engineering plays a crucial role. Common strategies to create defects in photocatalytic materials include heteroatom doping, formation of oxygen vacancies, and induction of lattice disorder [46, 105].

Black TiO_2_ has been demonstrated to exhibit outstanding NIR responsiveness through the introduction of vacancy defects into crystalline TiO_2_ subunits. Commonly employed reductive strategies include mechanical grinding with NaBH_4_ followed by calcination at 300–400°C [18], treatment under H_2_/N_2_ at 700°C [106], H_2_/aluminum at 500°C [107], and potassium treatment at 140°C [108]. Moreover, the introduction of oxygen vacancies into TiO_2_ can yield colored variants, such as blue and red TiO_2_, with absorption extending to the NIR region [109], offering additional pathways for tailoring NIR‐responsive photocatalytic materials.

In addition to oxygen vacancies, other defect engineering strategies, such as atomic doping and lattice disorder, remain largely unexplored in the context of NIR‐driven micro/nanomotors. These approaches offer promising potential for developing new photoactive materials capable of efficient NIR‐responsive propulsion. While defect engineering is an effective strategy for creating abundant active sites and improving performance, it may simultaneously result in impurity formation and further reduce the overall structural stability of photoactive materials [110]. Defects can function as recombination centers for photogenerated charge carriers and may accelerate photo‐corrosion under prolonged operation. In addition, achieving the precision required for custom atomic‐scale defects remains challenging for existing defect engineering methods [111]. Therefore, the design of NIR‐driven photocatalytic micro/nanomotors involves a critical trade‐off between improved light absorption and effective charge separation. Systematic operando spectroscopic studies (e.g., electron paramagnetic resonance (EPR), and transient absorption) correlating defect populations with propulsion performance will be pivotal to disentangle light‐harvesting enhancement from charge‐recombination losses.

#### 3.2.2 Photosensitization Strategies Using Dyes and Plasmonic Nanoparticles

Photosensitization, by coupling semiconductors with organic dyes or plasmonic nanoparticles, is another common approach to extend optical absorption into the NIR region. In this configuration, the photosensitizer absorb NIR photons and generate excitation electrons, which are then injected into the CB of the semiconductor to carry out the photo‐redox reactions and further provide driving force [112]. Therefore, exploring NIR‐absorbing dyes, including porphyrin derivatives [113], squaraine dyes, and borondipyrromethane (BODIPY) derivatives [114, 115], for building photoactive micro/nanomotors is of interest.

On the other hand, utilizing the localized surface plasmon resonance (LSPR) effect of plasmonic nanoparticles (such as Au, Ag and Cu) is another strategy to expand the light‐responsive region of micro/nanomotors. The detailed mechanism of plasmon has been discussed in many other excellent reviews [116, 117, 118]. Although plasmonic nanoparticles were initially used mainly in visible region, in recent years their absorption bands have been extended to farther wavelengths by careful tuning of their size and shape. For instance, Au nanorods/TiO_2_ exhibits absorption ranged from 700 to 1030 nm [119], Au nanorods/BiVO_4_ presents optical absorption ranged from 600 to 900 nm [120], and Ag nanoparticles/SiO_2_ displays absorption ranged from 400 to 820 nm depending on the size of Ag nanoparticles etc [121]. Hence, the combination of plasmonic metals with semiconductors provides a broad avenue still largely unexplored for designing NIR‐driven micro/nanomotors.

## 4 NIR‐Light‐Driven Photocatalytic Micro/Nanomotors for Therapeutic Treatments

**FIGURE 5:**
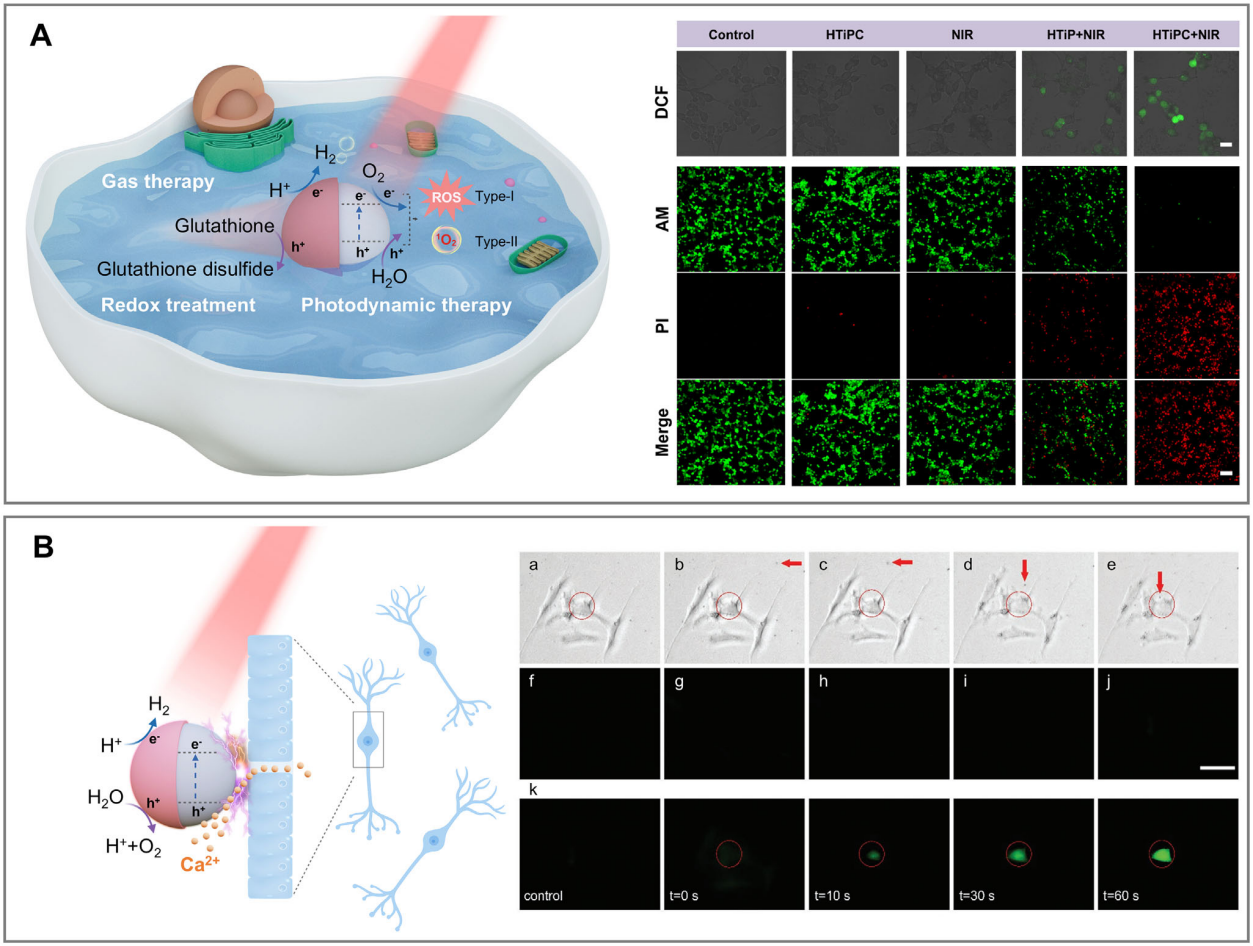
Schematic illustration and representative examples of photocatalytic NIR‐driven micro/nanomotors used for therapeutic treatments. (A) Gas therapy, redox treatment and photodynamic therapy. Adapted with permission.[18] Copyright 2024, American Chemical Society. (B) Modulation of single‐neuron activity. Adapted with permission.[123] Copyright 2021, John Wiley and Sons.The insets in (A) and (B) correspond to the in vitro antitumor therapy of Janus black H‐TiO_2‐x_‐based nanomotors, and the guided motion of the TiO_2_‐Au nanomotors toward the targeted RGC, respectively.

Photocatalytic micro/nanomotors offer distinct advantages for therapeutic approaches compared with other self‐propelled systems. Upon illumination, photogenerated electrons and holes can initiate redox reactions in biologically relevant media, enabling precise modulation of local chemical environments. Additionally, their inherent ability to produce ROS makes them excellent candidates for advanced medical applications, including PDT and antimicrobial photodynamic therapy (aPDT) [122]. These combined features make photocatalytic micro/nanomotors highly effective for targeted oxidative therapies that require controlled and localized reactivity. Figure 5 shows some representative examples of NIR‐driven micro/nanomotors for PDT, while also illustrating emerging opportunities in neural stimulation and cancer treatment. These applications will be discussed in more detail in the following subsections.

### 4.1 NIR‐Driven Photocatalytic Micro/Nanomotors for Photodynamic Therapy

In recent years, PDT has gained significant attention as a minimally invasive approach for the local destruction of diseased cells or malignant tumors. Great efforts have been devoted toward integrating micro/nanomotors into PDT systems, due to their capabilities of autonomous motion to the target sites with the assistance of magnetic guidance. Typically, the process of PDT consists of three essential components: the PSs, a light source and the presence of oxygen [124]. The broadly used strategy is to incorporate organic PSs into the micro/nanomotor structure to realize a combination of motion and function at the same time. Nevertheless, most of the conventional organic macromolecules‐based PSs, such as Photofrin, and indocyanine green (ICG) [125, 126], also suffer from insufficient photostability, rapid circulation time in the body and low absorption in NIR region, thus limiting the efficacy of PDT [127]. To overcome these limitations, inorganic nanoparticles have emerged as promising alternatives, offering tuneable photophysical properties through size control, morphology engineering, ionic doping, and defect modulation. As illustrated in Figure 5A, upon light irradiation, the charge carriers in inorganic materials can interact with surrounding species, such as H_2_O and O_2_, to produce ROS (e.g., cytotoxic singlet oxygen (^1^O_2_)), which then induce oxidative stress and eventually promote cell death [128].

To date, the most representative examples involve visible‐light‐triggered PDT using photocatalytic micro/nanomotors, such as hematite (α‐Fe_2_O_3_, E_g_: ca. 2.2 eV) and BiVO_4_ (E_g_: ca. 2.4 eV), which have been explored as PSs for ROS generation. For instance, dendrite‐shaped α‐Fe_2_O_3_ micromotors activated by blue light in the presence of H_2_O_2_, generate ROS that deplete intracellular glutathione (GSH) by 22%, contributing to prostate cancer cell treatment [129]. Similarly, concave BiVO_4_ micromotors have shown the ability to produce O_2_^•−^ and oxidative H_2_O_2_ species in H_2_O_2_ solution under solar light, which can be further used for disaggregating mature protein fibrils [130]. In this work, Thioflavin T (ThT) fluorescence assays were employed to monitor the transition from fibrils to monomers, revealing an overall ca. 83% decrease compared with native human serum albumin (HSA) fibrils without light exposure or H_2_O_2_ fuel.

In the context of NIR‐triggered PDT, a Janus Cu_2_O/Au nanomotor was reported to generate ROS under 808 nm irradiation, effectively inducing hepatocellular carcinoma cell death of 87% in combination with a photothermal therapy (PTT) effect [55]. The formation of Schottky junction between Cu_2_O and Au suppresses electron–hole recombination, thereby enhancing its ROS generation efficiency. However, the propulsion mechanism in this system primarily relies on the photothermal properties of Au rather than pure photocatalytic activity. Besides, black H‐TiO_2‐x_‐based nanomotors have been demonstrated to trigger intracellular oxidation and apoptosis by converting O_2_ into ^1^O_2_ under 808 nm laser irradiation [18], whereas their motion is still dependent on the enzymatic reaction of catalase (Figure 5A). Nevertheless, due to its unique optical and electronic properties, such defective TiO_2_ indeed holds great potential as fully photocatalytic NIR‐responsive systems capable of integrating both motion and therapeutic function. Analogous to this enzymatic‐driven propulsion and NIR‐activated PDT system, a nanomotor comprising a UCNP core and a Janus mesoporous silica shell loaded with chlorin e6 (Ce6) was designed [101]. Under 980 nm NIR laser irradiation, the Janus nanomotors emit visible light, which activates Ce6 to produce ROS and subsequently induces tumor cell apoptosis, reaching approximately 80% of efficiency.

While photocatalytic micro/nanomotors have demonstrated effective ROS generation for therapeutic purposes, several limitations still hinder their in vivo applicability. For example, many systems still depend on high concentration of H_2_O_2_ as chemical fuel or rely on photothermal effects for propulsion. In other cases, ROS generation was triggered by visible light, which suffers from poor tissue penetration. Moreover, most currently available inorganic photosensitizers exhibit poor biodegradability and unfavorable biological properties, including toxicity, non‐specific biodistribution, aggregation, and inefficient clearance [131]. These issues can result in prolonged retention in healthy organs and increase the risk of long‐term systemic toxicity, ultimately limiting their clinical translation. Therefore, a major challenge, and opportunity, for the field lies in developing photocatalytic micro/nanomotors capable of both autonomous motion and effective photodynamic activity under a single NIR light source. Achieving such system would mark a significant step toward minimally invasive, fuel‐free, and clinically relevant therapeutic technologies.

### 4.2 Emerging Therapeutic Applications of NIR‐Driven Photocatalytic Micro/Nanomotors

Recent studies suggest that the therapeutic use of photocatalytic micro/nanomotors may extend well beyond ROS generation. The ability of the light‐powered systems to interact with biological environments through localized electric fields, mechanical forces, or redox‐active processes, offers new directions in therapeutic applications. For instance, TiO_2_‐Au nanomotors, which demonstrate light‐induced mobility within vitreous tissue [132], have also been demonstrated to modulate single‐neuron activities through a locally generated electric field. In a related study [123], retinal ganglion cells (RGCs) served as a model to explore this effect. Upon optical guidance of the nanomotors toward the cells, the localized stimulation triggered the opening of calcium ion channels, resulting in calcium influx and subsequent neuronal activation. This was confirmed by the emergence of green fluorescence, indicating successful intracellular calcium accumulation (Figure 5B). Notably, these nanomotors moved in water via water splitting; however, the effect of H_2_ generation on cellular dynamics, e.g., ion channel activity or indirectly through its impact on redox balance was not considered in this study and remains an open question for future investigation.

Besides, it has been reported that H_2_ gas can selectively accumulate in mitochondria, where it contributes to cancer cell apoptosis by altering energy metabolism pathways [133]. Moreover, photoexcited electrons can reduce endogenous species such as H^+^/CO_2_/NO_3_^‒^ into H_2_/CO/NO, enabling gas therapy [134]. Simultaneously, photogenerated holes are capable of oxidizing intracellular GSH, a critical antioxidant that protects cells from oxidative damage, into glutathione disulfide (GSSG) to disrupt the antioxidant microenvironment, thus enhancing the efficacy of ROS treatment and contributing to cancer therapy [133]. These examples highlight the unique therapeutic potential of photocatalytic micro/nanomotors, driven by their unique capacity to generate electron–hole pairs, produce functional gases such as H_2_, and modulate the biochemical environment through oxidation of vital cellular components.

Table 3 summarizes key examples of photocatalytic micro/nanomotors previously developed for various purposes, detailing their photostability, NIR activation strategies, and potential biomedical functions. Although many of the reported systems were not originally designed for therapeutic use, we outline how their structural and functional features can be adapted or optimized for biomedical applications. In addition, we propose a set of strategies to enhance their performance specifically in the NIR region, including the formation of heterostructures [135, 136, 137, 138], incorporation with UCNPs [139, 140], alteration of materials structure [138, 141, 142, 143, 144], decoration of plasmonic metals [145], and metal doping [146, 147]. These approaches offer pathways to tailor the optical and catalytic properties of micro/nanomotors, enabling the application in targeted cancer therapy, PDT, antimicrobial treatments, and neural stimulation.

**TABLE 3:** Representative examples of the main photocatalytic micro/nanomotors developed for therapeutic applications.

Micromotor type	Bandgap (eV)	Photostability	Strategies for achieving NIR photoresponse	Therapeutic potential	Refs.
WS_2_	1.4	Low	—	Effective in photothermal therapy but requires addition of toxic chemical fuels for propulsion	[47]
Cu_2_O	2.1	Low	Incorporation with GO, rGO, PbS, Ag	Effective in glucose‐rich biological media with potential for in vivo applications	[48, 49, 148]
Si	1.1	Moderate	Formation of p‐n heterojunction	Suitable for water splitting in targeted cancer therapy with good biocompatibility	[69]
TiO_2_	3.2	High	Black hydrogenation, incorporation with NaYF_4_:Yb^3+^/Tm^3+^	Potential for neural stimulation and photodynamic therapy via ROS generation	[18]
Fe_2_O_3_	2.2	Moderate	Incorporation with NaYF_4_:Yb^3+^/Er^3+^, MoS_2_	Effective movement in high‐ionic environments, and potential for photothermal therapy and theranostics	[129]
BiVO_4_	2.4	High	Decoration of plasmonic metals: Ag, Metal doping: Er^3+^, Yb^3+^, Tm^3+^	Potential for protein disaggregation and antimicrobial photodynamic therapy through ROS generation	[130]
PHI (Poly‐heptazine imide)	3.0	High	Tailoring defect ionization energy	Stable in high‐ionic environments, potential for diverse biomedical applications	[149]
Ag_3_PO_4_	2.1	Low	Incorporation with NaYbF_4_:Tm^3+^	Effective in glucose‐rich biological media with promise for antimicrobial photodynamic therapy	[150]
C_3_N_4_	2.8	High	Incorporation with NaYF_4_:Yb^3+^/Tm^3+^, CuS	Useful in bioimaging, effective in glucose‐rich biological media and biocompatible	[151]
CdSe QDs	1.7	Low	—	Effective movement in blood with biocompatible fuel (glucose) and suitable for water splitting in targeted cancer therapy	[152]

## 5 Challenges and Opportunities for Photocatalytic Micro/Nanomotors in Complex Biological Environments

**FIGURE 6:**
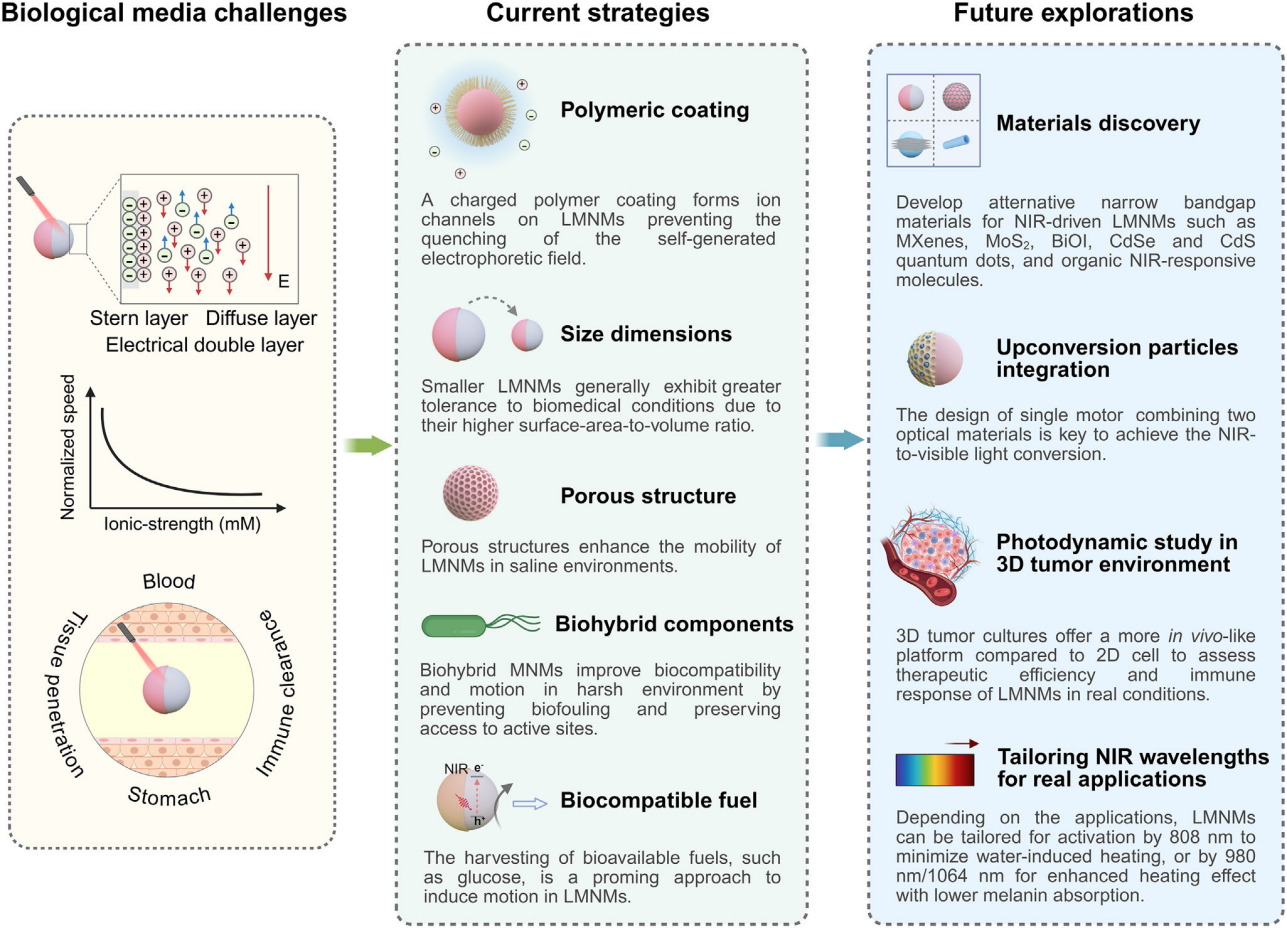
Schematic illustration of the main challenges encountered when using NIR‐driven photocatalytic micromotors in various biological environments, along with common strategies and future explorations to enhance their motion performance. LMNMs: light‐driven micro/nanomotors.

One of the most promising applications of NIR‐driven photocatalytic micro/nanomotors is in the biomedical field. However, their effective deployment in therapeutic treatments is required to overcome significant challenges associated with complex biological environments, including highly ionic media, extreme pH conditions, elevated viscosity, and biochemical interference. Moreover, immune system clearance remains a significant challenge, as immune responses can quickly neutralize or eliminate micro/nanomotors before they reach their target, diminishing their therapeutic effectiveness. Given that these issues represent common challenges for micro/nanomotors driven by light across all wavelengths, this section will transcend the limitations of NIR light source. It offers a broader perspective on existing technological constraints and outlines forward‐looking strategies for improving these limitations. Figure 6 presents the existing challenges, currently utilized strategies (such as polymetric coating, size dimensions, porous structure, biohybrid components, biocompatible fuel), and potential future research directions for photocatalytic micro/nanomotors in biological applications, such as materials discovery, upconversion particles integration, photodynamic study in 3D tumor environment, tailoring NIR wavelengths for real‐world scenarios. The detailed discussion is given in the following sections.

### 5.1 Motion in Biocompatible Fuels

The application of photocatalytic micro/nanomotors in biological environments presents exciting opportunities yet poses significant challenges due to the complex nature of physiological fluids such as blood, mucus, and interstitial fluids. These media are characterized by high viscosity and the presence of proteins, cells, and other macromolecules that can interfere with light penetration and hinder propulsion efficiency [153, 154]. A critical aspect of advancing their biomedical potential lies in developing micro/nanomotors that can move effectively in such conditions while avoiding the use of toxic chemical fuels.

Recent efforts have focused on enabling photocatalytic micro/nanomotors to utilize biologically relevant components, such as endogenous H_2_O_2_, glucose, organic acid, or other redox‐active species as fuel sources [155, 156, 157]. Among these biologically relevant fuels, glucose stands out as an ideal candidate due to its natural abundance in the human body, particularly in the bloodstream, liver, and muscle tissues, where it plays a vital role in metabolism. Its ubiquity allows glucose to be a promising fuel source for powering photoactive micro/nanomotors using endogenous energy. A notable example is the development of Cu_2_O micromotors integrated with nitrogen‐doped carbon nanotubes (N‐CNTs) [156]. The N‐CNTs could capture electrons photogenerated from Cu_2_O side, thereby enhancing electron‐hole pair separation and improving the overall motion efficiency. In the presence of glucose, these micromotors showed improved self‐propulsion compared to their activity in pure water. The enhanced movement was attributed to a diffusiophoretic mechanism, where photogenerated holes decompose glucose into various organic compounds and protons, which are subsequently reduced to H_2_ by the photogenerated electrons. However, the exact identity of the oxidation products was not experimentally confirmed. Other photocatalytic micromotors, such as Ag_3_PO_4_ and C_3_N_4_‐based derivates [150, 151], have also demonstrated the ability to self‐propel in the presence of glucose. These results suggest that a wide range of photocatalytic materials, regardless of their specific optical properties, offer potential for utilizing biocompatible fuels due to their inherent capacity to oxidize organic compounds under light irradiation. In particular, a visible light‐driven Janus CdTe QDs/Fe_2_O_3_ micromotor exhibited effectively self‐propulsion in human blood serum and whole blood when supplemented with glucose [158]. However, the glucose concentration used in this study (200 mM) was relatively higher than physiological levels in the human body, which are typical around 10 mM [159]. It is noteworthy to mention that using lower, more physiologically relevant concentrations are essential to evaluate the practical applicability of glucose‐powered photocatalytic micro/nanomotors.

### 5.2 Motion in Highly Ionic and Conductive Environments

Photocatalytic micro/nanomotors typically move through phoretic mechanisms, such as self‐electrophoresis and self‐diffusiophoresis, depending on their material configuration [160]. As a result, in highly ionic environments, e.g., body fluids, the motion may be significantly hindered or completely quenched. As the ionic strength increases, the Debye length shortens, thus reducing the local electrical field that drives the movement and ultimately leading to minimal or halted motion [38]. The Median Effective Ionic Strength (*EI_50_*) is employed to assess how the ionic strength of a medium affects the movement of micro/nanomotors. *EI_50_* refers to the ionic strength at which the migration speed of a micro/nanomotor is reduced by 50% compared to its speed in a solution without added electrolytes [161]. This metric helps quantify the impact of ionic concentration on propulsion efficiency, which is a useful for determining how well light‐driven micro/nanomotors can operate in ion‐rich environments. Common strategies to overcome motion limitations in high‐ionic environments include surface functionalization with polymeric coatings, optimizing the size of micro/nanomotors, and incorporating porous structures into their design (Figure 6).

#### 5.2.1 Polymer Coating

The coating of light‐driven micro/nanomotors with a highly charged polymer creates an ion‐conductive channel on their surface, which further contributes to preventing the quenching of the self‐generated electrophoretic field. For instance, the application of sulfonated polystyrene (SPS) coatings onto silicon‐based micromotors or conductive polyaniline coating on TiO_2_‐based micromotors has been shown to significantly improve surface conductivity [161, 162]. This enhancement reduces excessive ion interference that can disrupt the Debye layers in electrolyte solutions, which are crucial for efficient propulsion. As a result, these surface‐modified photoactive micromotors were able to self‐propel in NaCl solutions at concentrations of 0.3 and 10 mM, respectively. However, it is important to note that the polymer coatings can also slow down the speed of the micromotors. This drawback can be mitigated by using higher concentrations of chemical fuel to compensate for the drag force induced by the coating.

#### 5.2.2 Size Dimensions

The performance of micro/nanomotors in highly ionic environments is also strongly influenced by their size and geometry. Smaller micro/nanomotors generally exhibit greater tolerance to these conditions due to their higher surface area‐to‐volume ratio, which improves the interaction with the surrounding medium and further achieve more efficient movement in ion‐rich media. This effect is especially pronounced by nanomotors with dimensions approaching tens of nanometers or smaller. So far, lowering the scale dimensions to enhance motion efficiency has been demonstrated not only in Si‐based nanomotors but also in Fe_2_O_3_ nanomotors. In fact, the latter showed a *EI_50_* of 30.2 mM [163], significantly surpassing the performance of polyelectrolyte‐coated ion‐tolerant photocatalytic nanomotors (*EI_50_* of 3.82 mM). Such enhanced ion tolerance is likely due to the smaller size of Fe_2_O_3_ nanomotors, with a diameter of 140 nm and an average length of 230 nm, compared to Si‐based nanomotors with a diameter of 400 nm and length of 2 µm [161]. Additionally, these Fe_2_O_3_ nanomotors exhibited collective clustering interactions in high‐salt environments, such as 150 mM NaCl, which is comparable to the electrolyte concentrations found in human fluids. Besides, nanoscale dimensions also enable nanomotors to effectively bypass biological barriers, improving their potential for targeted delivery and therapeutic use. Consequently, the development of nanoscale motors with controllable functionality is highly desirable for practical applications.

#### 5.2.3 Porous Structures

The design of porous structures plays a crucial role in enhancing micro/nanomotor motility in saline environments. For instance, Janus Au/BiOI micromotors, based on porous microparticles, have shown propulsion in 1 mM NaCl solution upon photoactivation [57]. Beyond structural porosity, incorporating optoionic properties in photocatalytic micro/nanomotors offers an interesting approach to improve the motion of these devices in ionic and biological media. In optoionic systems, light modulates ion flow and conductivity, making these properties highly advantageous for boosting the performance of photoactive micro/nanomotors. Light‐driven poly‐heptazine imide (PHI) micromotors, for instance, maintained consistent movement in high molarity salt solutions (up to 5 M NaCl) and biological fluids, such as buffer solutions, cell culture media, and diluted blood, without requiring additional fuels [149]. This enhanced motion capability was attributed to optoionic effects, which preserve speed and ion tolerance while optimizing photocatalytic activity for effective propulsion. However, the potential of this approach for NIR‐driven photocatalytic micromotors, beyond the UV‐responsive carbon nitride derivatives, remains largely underexplored. Further research is essential to assess the applicability of optoionic effects in NIR‐driven micromotors. In addition to designing micro/nanomotors with intrinsic porosity, coating their surfaces with a porous layer offers another effective strategy to enhance the ion‐resistance. For example, the porous metal‐organic framework (MOF) ZIF‐8 has been applied as a coating on silicon micromotors, where it functions as a surface scaffold that helps to preserve the conductive “Debye layers” [164]. By optimizing both MOF layer and geometry factor, the *EI_50_* of the modified micromotors was enhanced by 266 times compared to uncoated micromotors under 980 nm NIR light. This study expands the potential of existing NIR‐driven micro/nanomotors for advanced biomedical applications by simply coating porous materials.

### 5.3 Motion in Extreme pH Conditions

Micro/nanomotors used in biomedical applications often encounter environments with extreme pH values, such as the acidic conditions of the stomach or the slightly basic conditions of the intestines. Variations in the pH of the surrounding medium can alter the surface charge of photocatalytic materials, which is closely tied to their point of zero charge (PZC). The PZC refers to the pH at which the net surface charge of particles becomes neutral and is influenced by the intrinsic properties of the materials and the surrounding liquid environment. In harsh pH environments, the environmental pH deviates from the PZC, resulting in surface charge changes that lead to electrostatic repulsion between micro/nanomotors and the fuel or surrounding ions, hindering activity or blocking adsorption sites. In some cases, however, favorable pH changes can enhance micro/nanomotor activity, requiring careful optimization based on application conditions [165, 166]. For instance, BiVO_4_ micromotors have demonstrated improved motion in highly alkaline conditions (pH 10), which is due to the change in the surface charge that enhances chemical fuel adsorption [167]. Additionally, photocatalytic micromotors can oxidize organic acids, such as citric acid [168], and malic acid [59, 157], which are naturally present in the human body. The shorter the aliphatic organic acids the easier it will be decomposed [169]. Therefore, in a similar way to glucose, organic acids can also act as sustainable chemical fuels, driving the photocatalytic propulsion of the micro/nanomotors through oxidation processes.

Other strategies to achieve motion of photocatalytic micro/nanomotors in more acidic environments (pH ≤ 3) often involve the incorporation of reactive materials such as Zn or Mg [170, 171, 172], which can react with HCl, generating H_2_ gas bubbles that drive propulsion. However, since these metals are consumed during the reaction, they provide only temporary motion, highlighting the need for alternative strategies that enable sustained or reusable propulsion in such environments. An appealing strategy involves the development of biohybrid micro/nanomotors using naturally occurring algae, which exhibit a strong tolerance to highly acidic environments, even at pH levels as low as 1 [173]. These biohybrid systems can potentially serve as scaffolds for loading photoactive nanoparticles, enhancing their functionality with a photocatalytic response [174].

## 6 Summary and Outlook

Photocatalytic micro/nanomotors represent a rapidly advanced class of light‐driven active systems, with growing relevance in biomedical applications. The use of NIR light to activate their motion confers significant advantages, such as deep tissue penetration, less pronounced scattering, lower phototoxicity, and minimal interaction with other biological components. Importantly, NIR‐responsive photocatalytic micro/nanomotors can carry out precise chemical processes, including localized redox reactions and ROS production, while minimizing damage to surrounding healthy tissue. Such targeted capabilities make them promising candidates for non‐invasive therapeutic applications, ranging from drug‐free treatments to PDT and other redox‐based strategies.

Recent progress in heterostructure engineering has broadened the functional capabilities of NIR‐driven photocatalytic micro/nanomotors by improving NIR light harvesting, charge separation and photostability under physiologically relevant conditions. From a material‐design perspective, a careful control of band gap alignment is necessary to match the redox potential of the resulting heterojunction with the desired bio‐catalytic reaction. At the same time, biosafety and biostability impose critical constrains on material choice. In particular, narrow‐bandgap materials such as Cu_2_O, CdS‐based systems, PbS, and BP, raise concerns related to photostability or long‐term biocompatibility in biological environments. Possible strategies to mitigate these limitations include the use of biocompatible surface coatings, including hydrogels, liposomes, and fibrin. For inorganic/UCNP hybrid systems, attention should extend beyond the selection of dopant pairs, as the surface characteristics of the support materials, particularly surface roughness, also play a crucial role in enhancing UCNP attachment stability and facilitating interfacial electron transfer.

Among defect‐engineering strategies, reduced TiO_2_ emerges as a particularly attractive material, as it combines extended light absorption with the well‐established biocompatibility and chemical robustness of TiO_2_. In addition, combining defect engineering with template‐assisted synthesis offers a versatile route to produce TiO_2_ hollow spheres with controllable dimensions, which is highly relevant for optimizing light‐matter interactions and propulsion behavior.

On the other hand, materials such as Cu_2_O‐based heterostructures and n‐Si/p‐Si systems have become reference points in developing NIR‐driven photocatalytic micromotors. It is noteworthy that currently the reliance on H_2_O_2_ or 1,4‐benzoquinone/hydroquinone consumption and complex fabrication process, such as photolithography, still complicate their full applicability. Particularly, quinone‐based compounds are known to pose cytotoxic risks in redox reactions. For instance, in biological systems, quinones can be reduced by cellular reductases to form semiquinones or hydroquinones, which may act as oxidizing or dehydrogenating agents [175]. Therefore, the use of such redox couple for activation of NIR‐responsive micro/nanomotors in biological environments requires careful assessment and optimization of chemical concentration to ensure both safety and efficacy. Besides, numerous materials still remain unexplored, signalling an opportunity for the research community to dive deeper into designing novel configurations of NIR‐photocatalytic micro/nanomotors. For example, 2D materials, such as MXenes [176, 177], with their high electrical conductivity, large surface area, and narrow bandgaps [178], are particularly well‐suited for coupling with wide‐bandgap semiconductors to form efficient NIR‐responsive platforms. In parallel, the exploration of NIR‐responsive molecules, such as phthalocyanines and porphyrins [179], which can be fine‐tuned by altering metal centers, also presents an attractive strategy for expanding optical absorption and improving ROS‐mediated therapeutic functionality of light‐driven micro/nanomotors [180, 181, 182].

When it comes to motion in biological fluids, photocatalytic micro/nanomotors have already shown considerable promise in navigating challenging environments. Size reduction has proven to be effective in achieving movement in highly ionic environments, and several systems have demonstrated stable performance across a wide range of pH levels, including organic acids and basic media. More impressively, emerging studies indicate that these micro/nanomotors are capable of crossing biological barriers, such as cellular membranes and the vitreous tissue of the eye, demonstrating their potential for operation in biologically relevant environments.

While progress has been achieved, there are still challenges to address, particularly in improving the biocompatibility of photocatalytic micro/nanomotors. A critical factor is the formation of a protein corona, a layer of adsorbed biomolecules on the micro/nanomotor surface upon exposure to biological fluids. The resulting protein corona layer can diminish motion efficiency and, in some cases, provoke undesirable immune responses. One promising strategy to overcome the immune clearance is to coat the micro/nanomotors with cell membranes, derived from leukocytes [183], red blood cells [184, 185], or platelets [186], enabling biomimetic camouflage that facilitates immune evasion and significantly prolong the circulation time in vivo. However, it is critical to understand how surface functionalization affects both the motion capabilities and photoactivity of the micro/nanomotors, especially when they are coated with cell membranes. Preliminary studies suggest that biocompatible polymer coatings do not directly affect their photocatalytic motion [162]. However, systematic investigations are still required to assess the long‐term effects, including motion performance under low‐energy conditions such as NIR light irradiation, to ensure sustained functionality over extended illumination periods. In parallel, comprehensive cytotoxicity evaluations across multiple cell types are essential to identify the most suitable material components for specific biomedical applications.

Regarding therapeutic applications, NIR‐driven micro/nanomotors present unique advantages for PDT, such as the ability to generate ROS and interact with surrounding fluids through redox processes. For instance, upon NIR light irradiation, the micro/nanomotors generate H_2_ and O_2_ by photoactivated water splitting, which can disrupt cancer cell metabolism and enhance ROS levels in hypoxic tumor environments, respectively [187, 188]. In addition, the oxidation of GSH by the photogenerated holes can weaken antioxidant defenses in cancer cells and promote apoptosis. In this regard, further studies should focus on improving the accumulation of NIR‐driven photocatalytic micro/nanomotors in targeted regions though surface functionalization, such as with folic acid [189, 190], to enhance their therapeutic effectiveness in PDT. The long‐term in vivo biosafety of micro/nanomotors requires further investigation. For synthetic inorganic micro/nanomotors, it is critical to assess their ultimate fate after task completion to identify potential side effects, such as their degradation pathways, byproducts, and associated in vivo toxicity [191]. For micro/nanomotors that cannot self‐degrade, incorporating biocompatible magnetic materials into their design not only enhances navigation capabilities but also allows for their recovery after completing specific tasks. Moreover, testing the NIR‐light driven micro/nanomotors in 3D tumor cultures, which better mimic in vivo conditions than traditional 2D cells, will be essential for assessing therapeutic efficiency and immune response [192, 193], providing more reliable and relevant results for preclinical testing.

On the other hand, the selection of appropriate NIR wavelengths is also an important parameter to ensure effective operation of photocatalytic micro/nanomotors, tailored to the specific applications [182, 194]. While 808 nm is a commonly used wavelength due to its superior tissue penetration compared to red light, this wavelength has relatively low water absorption, making it suitable for applications that require minimal heating. By exploring other wavelengths such as 980 nm, which combines a stronger heating effect due to higher water absorption [195], or 1064 nm, which minimizes superficial melanin absorption, more versatile and efficient micro/nanomotors could emerge. In this sense, the design of UCNPs/photocatalytic micro/nanomotors is an interesting approach with great possibilities to extend their therapeutic applications to the second biological window.

In summary, NIR‐driven photocatalytic micro/nanomotors are expected to play a pivotal role in shaping the next generation of biomedical technologies. Realizing their full potential, however, will require a highly interdisciplinary effort that bridges materials science, nanotechnology, engineering, and biomedical research. We encourage the scientific community to intensify efforts toward the development of novel NIR‐responsive micro/nanomotor designs and to rigorously evaluate their performance in biologically relevant models. With continued innovation and collaboration, NIR‐light driven micro/nanomotors have the potential to move beyond proof‐of‐concept demonstrations and emerge as transformative tools for precision therapy and minimally invasive, light‐driven interventions.

## Conflicts of Interest

The authors declare no conflict of interest.
